# Phosphate Acquisition in Protozoan Parasites: Transport Systems, and Extracellular Phosphate-Releasing Enzymatic Activities

**DOI:** 10.3390/ijms27093707

**Published:** 2026-04-22

**Authors:** Anita Leocadio Freitas-Mesquita, José Roberto Meyer-Fernandes

**Affiliations:** 1 Centro de Ciências da Saúde, Instituto de Bioquímica Médica Leopoldo de Meis, Universidade Federal do Rio de Janeiro, Rio de Janeiro 21941-590, RJ, Brazil; 2 Instituto Nacional de Ciência e Tecnologia em Biologia Estrutural e Bioimagem, Rio de Janeiro 21941-590, RJ, Brazil

**Keywords:** protozoan parasites, phosphate acquisition, ectophosphatases, phosphate transport systems, phosphate-limiting conditions

## Abstract

Inorganic phosphate (Pi) is an essential nutrient required for energy metabolism, macromolecule biosynthesis, and signal transduction. Protozoan parasites are exposed to pronounced fluctuations in nutrient availability throughout their life cycles and therefore rely on adaptive strategies to secure phosphate from diverse host and environmental niches. This review summarizes current knowledge on phosphate acquisition mechanisms in protozoan parasites, with emphasis on membrane transport systems and extracellular phosphate-releasing enzymatic activities. Phosphate transport systems energized by proton or sodium gradients have been functionally characterized in several protozoan species, and in many cases phosphate uptake capacity is modulated by extracellular Pi availability. In addition, ectophosphatases expressed at the parasite surface contribute to phosphate acquisition by hydrolyzing extracellular phosphorylated substrates and releasing inorganic phosphate that can be subsequently internalized and metabolically utilized. Although phosphate-dependent regulation of ectophosphatase activity has been demonstrated in a more limited number of species, available evidence supports a functional interplay between extracellular phosphate scavenging and transmembrane transport, particularly under phosphate-limiting conditions. Despite these advances, the molecular mechanisms underlying phosphate sensing and regulatory coordination in protozoan parasites remain poorly understood. This review provides a comparative overview of phosphate transport systems and extracellular phosphate-scavenging enzymes in protozoan parasites, highlighting current evidence and remaining knowledge gaps.

## 1. Introduction

Inorganic phosphate (Pi) is a central metabolite required for a wide range of cellular processes. For parasitic protozoa, whose life cycles involve transitions across diverse host environments and nutrient landscapes, efficient strategies for phosphate acquisition are critical for survival, proliferation, and pathogenicity. This review summarizes current knowledge on phosphate acquisition mechanisms in protozoan parasites, with a focus on membrane transport systems and extracellular phosphate-scavenging enzymes. We discuss evidence supporting functional integration between these processes under phosphate-limiting conditions and highlight major gaps in our understanding of phosphate sensing and regulatory pathways in these organisms.

## 2. Phosphate Availability and Adaptive Acquisition Strategies in Microorganisms

**Figure 1:**
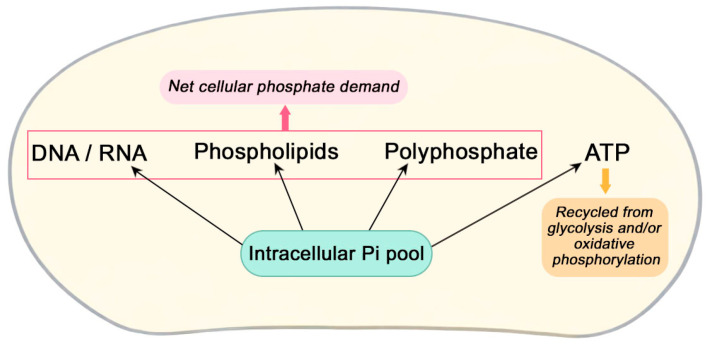
Intracellular phosphate utilization and its major metabolic sinks. Intracellular phosphate (Pi) participates in both metabolic and biosynthetic processes. While phosphate involved in ATP turnover is continuously recycled within the cell, the net phosphate demand is primarily driven by its incorporation into macromolecules such as nucleic acids, phospholipids, and polyphosphate reserves. These components function as major intracellular phosphate sinks, requiring sustained phosphate uptake to support cellular growth and structural maintenance.

Pi is an essential nutrient for all living organisms, as it participates in a wide range of cellular processes, including energy metabolism, cellular signaling, and the biosynthesis of nucleic acids, phospholipids, and phosphorylated metabolic intermediates. Phosphate participates in energy metabolism through ATP turnover, although this pool is largely recycled within the cell. The net cellular demand for phosphate is primarily driven by its incorporation into macromolecules such as nucleic acids, phospholipids, and polyphosphate reserves, which function as major intracellular phosphate sinks (Figure 1). To support the structural and biosynthetic requirements of the cell, organisms have evolved tightly controlled mechanisms to ensure adequate phosphate acquisition, storage, and mobilization in response to environmental availability [1,2].

Protozoan parasites are exposed to marked fluctuations in nutrient availability throughout their life cycles, as they alternate between distinct hosts, tissues, and cellular environments. During infection, they depend on host-derived resources while competing with host cells for essential metabolites, imposing strong selective pressure on the evolution of efficient and adaptive nutrient acquisition strategies [3,4].

Upon phosphate limitation, eukaryotic microorganisms use a set of adaptive strategies to maintain Pi homeostasis [5]. These include the mobilization of extracellular phosphate sources through secreted and surface-associated phosphatases; the controlled release of intracellular phosphate reserves such as vacuolar polyphosphate; and, under prolonged starvation, the recycling of Pi from essential cellular constituents, such as nucleotides and phospholipids. Phosphate starvation ultimately leads to growth retardation, as cells must prioritize the reallocation of their limited intracellular Pi pool toward biosynthetic processes essential for survival rather than sustained proliferation [2].

Acidocalcisomes are electron-dense acidic organelles enriched in polyphosphate as well as inorganic and organic cations, whose membranes harbor multiple channels, pumps, and transporters. These organelles are evolutionarily conserved, being found in both bacteria and eukaryotes, and have been characterized in greatest detail in trypanosomatid parasites [6]. Acidocalcisomes provide an intracellular reserve that enables parasite survival under sudden Pi starvation, facilitating an ordered transition into a robust quiescent state. The mobilization of polyphosphate reserves primarily serves as a buffering mechanism, enabling short-term adaptation and delaying the activation of more deleterious phosphate-recycling pathways [2]. On the other hand, phosphate acquisition from extracellular sources represents an adaptive strategy to sustain metabolic activity without depleting internal stores.

Given the limited permeability of biological membranes to Pi, protozoan parasites rely on specialized transport systems to ensure phosphate uptake. These systems predominantly operate through secondary active transport mechanisms driven by electrochemical ion gradients, enabling phosphate acquisition across a wide range of physiological conditions. In eukaryotes, Pi uptake is mediated by distinct transporter families, including PiT-type Na^+^-dependent transporters, which are primarily associated with basal phosphate homeostasis, and PHS-type H^+^-coupled transporters, which are frequently induced under phosphate-limiting conditions and functionally linked to extracellular phosphate scavenging mechanisms [2].

The coexistence of high- and low-affinity phosphate transport systems allows cells to maintain phosphate uptake over a broad range of environmental concentrations. As external phosphate levels decline, the reduced efficiency of low-affinity transporters leads to intracellular phosphate depletion. Across microorganisms, phosphate depletion triggers adaptive responses that commonly involve increased expression of high-affinity uptake systems and phosphate-scavenging enzymes, contributing to the maintenance of phosphate homeostasis [7,8]. These variations in extracellular phosphate availability trigger regulatory responses involving changes in gene expression and protein activity. The phosphate signal-transduction pathway, known as the PHO pathway, has been extensively characterized in model organisms such as yeast and plants [9,10].

In the model unicellular organism *Saccharomyces cerevisiae*, phosphate acquisition relies on transport systems with distinct affinities, including high-affinity transporters (Pho84 and Pho89), induced under phosphate limitation, and low-affinity transporters (Pho87 and Pho90), which operate preferentially under phosphate-replete conditions. Fluctuations in extracellular phosphate ultimately impact intracellular phosphate pools, leading to modulation of the cyclin-dependent kinase complex Pho80–Pho85. Under phosphate scarcity, inhibition of this complex by Pho81 allows the activation and nuclear localization of the transcription factor Pho4, which acts cooperatively with Pho2 to induce a transcriptional program dedicated to phosphate acquisition. This program includes the expression of high-affinity phosphate transporters and secreted acid phosphatases, such as Pho5, Pho11, and Pho12, which enable the liberation of inorganic phosphate from extracellular phosphorylated compounds. Together, these mechanisms exemplify how extracellular phosphate functions not only as a nutrient but also as a regulatory signal that coordinates transporter activity and phosphatase-mediated phosphate scavenging [2,11,12].

In addition to its role as a high-affinity phosphate transporter, Pho84 has also been proposed to function as a phosphate transceptor, linking extracellular phosphate availability to intracellular signaling. Under phosphate-limiting conditions, when Pho84 is present at the plasma membrane, binding of extracellular phosphate can trigger signaling responses independently of net phosphate uptake. This activity has been associated with activation of the protein kinase A (PKA) pathway, a key regulator of metabolic reprogramming and cell cycle progression [12,13,14,15].

This dual role highlights phosphate transport systems not only as mediators of nutrient uptake but also as key regulatory nodes integrating environmental sensing with metabolic responses. Because these systems couple phosphate acquisition to adaptive signaling pathways, they represent particularly attractive targets for pharmacological intervention. In *Candida albicans*, the high-affinity phosphate transporter Pho84 has been shown to be required for full virulence and resistance to oxidative stress, with pho84-deficient strains displaying increased susceptibility to host immune defenses [16]. Pharmacological inhibition of Pho84, including by small molecules such as foscarnet, induces intracellular oxidative stress and impairs fungal fitness [16]. In addition, combined targeting of phosphate transport and cell wall biosynthesis has been proposed as a synergistic antifungal strategy [17]. Importantly, fundamental differences between fungal and human phosphate homeostasis suggest that phosphate transport systems may represent selective and promising targets for antifungal intervention [17].

In several pathogenic bacteria, including *Escherichia coli*, *Pseudomonas aeruginosa*, and *Salmonella enterica*, phosphate homeostasis is regulated by the Pho regulon, which links environmental phosphate sensing to the expression of virulence-associated genes. Central to this system are phosphate transporters such as the high-affinity PstSCAB complex, required for adaptation to phosphate-limited conditions during infection. Disruption of Pho regulon components, including PhoB and PhoR, impairs virulence traits, biofilm formation, and host colonization, highlighting the tight connection between phosphate acquisition and bacterial pathogenicity and reinforcing their potential as targets for antimicrobial intervention [18].

Although the existence of a canonical PHO signaling pathway in trypanosomatids remains to be fully elucidated, available evidence indicates that phosphate starvation enhances both Pi uptake and ectophosphatase activity in several species, as discussed in the following sections.

## 3. Phosphate Transport Systems in Protozoan Parasites

Early physiological studies had already indicated that several protozoa depend on an external supply of inorganic phosphate to sustain normal growth, but the identity of the transporters mediating Pi uptake remained unknown. A major advance in this field occurred in 2006, with the first molecular description of a plasma membrane phosphate transporter in the intraerythrocytic parasite *Plasmodium falciparum* [19]. This transporter, designated PfPiT, was shown to belong to the PiT family and to mediate phosphate uptake across the parasite plasma membrane, thereby providing a direct molecular link between extracellular phosphate availability and parasite growth. The identification of PfPiT established phosphate transport as a defined and regulated process in protozoan parasites and paved the way for subsequent studies aimed at elucidating the diversity, regulation, and physiological relevance of phosphate transport systems in these organisms [19].

In *Toxoplasma gondii*, phosphate uptake was shown to rely on a single sodium-dependent phosphate transporter, designated TgPiT, that localizes predominantly to the parasite plasma membrane, as well as to endosomal compartments and cytoplasmic vesicles. Genetic ablation of TgPiT resulted in a severe impairment of phosphate import, leading to reduced intracellular Pi and polyphosphate levels, profound defects in parasite replication, and markedly attenuated virulence in vivo. In addition to its role in phosphate acquisition, loss of TgPiT caused pronounced alterations in cellular homeostasis, including reduced cell volume, abnormal vacuolar morphology, disrupted calcium storage, and extensive accumulation of acidocalcisomes. Together, these findings reveal a tight functional connection between phosphate transport, intracellular phosphate storage, and the maintenance of parasite physiological integrity [20].

A direct functional link between phosphate uptake and energy metabolism has been demonstrated in anaerobic and microaerophilic protozoa such as *Giardia duodenalis* [21] and *Tritrichomonas foetus* [22]. In *G. duodenalis*, a putative plasma membrane H^+^:Pi symporter (GdPho84) mediates active phosphate uptake, and inhibition of this process leads to reduced parasite proliferation and intracellular ATP levels, revealing a tight coupling between phosphate acquisition and glycolytic ATP production [21]. Similarly, in *T. foetus*, extracellular phosphate release by surface-associated ectophosphatases and subsequent Pi uptake are functionally linked to ATP synthesis, as interference with either enzymatic activity or phosphate transport results in decreased ATP availability and impaired growth [22]. Together, these findings highlight phosphate uptake as a key determinant of bioenergetic homeostasis in these parasites.

The first mechanistic characterization of phosphate transport across the plasma membrane in trypanosomatids was carried out in *Trypanosoma rangeli*. In this species, two distinct uptake modes were identified: one associated with Na^+^-ATPase activity and another linked to H^+^-ATPase function, both contributing to phosphate acquisition during parasite development [23]. Subsequent studies extended these observations to *Leishmania infantum*, in which a high-affinity H^+^/Pi cotransporter was identified [24]. In *Leishmania donovani*, the presence of both Na^+^-dependent and H^+^-dependent Pi transport systems was later reported, supporting the existence of conserved phosphate uptake mechanisms within the genus *Leishmania* [25]. A similar dual transport system was subsequently characterized in *Phytomonas serpens*, where high-affinity phosphate uptake driven by both Na^+^ and H^+^ gradients was described, further reinforcing the notion that trypanosomatids commonly rely on multiple, partially redundant phosphate transport systems to ensure efficient Pi acquisition under diverse environmental conditions [26].

The molecular characterization of phosphate uptake revealed a more complex scenario in *Trypanosoma brucei*. A gene initially annotated as a putative phosphate transporter based on sequence similarity to the yeast high-affinity transporter Pho84 was subsequently identified as a proton-coupled *myo*-inositol transporter, designated TbHMIT. Functional expression studies demonstrated that TbHMIT mediates H^+^-dependent *myo*-inositol uptake but does not directly transport inorganic phosphate. Nevertheless, genetic knockdown of TbHMIT resulted in a marked reduction in Pi uptake and impaired parasite growth, indicating an indirect functional link between *myo*-inositol transport and phosphate acquisition. In addition, excess extracellular *myo*-inositol inhibited Pi uptake, further supporting the existence of a regulatory or functional interaction between these processes. Although the identity of the genuine phosphate transporter in *T. brucei* remains unresolved, these findings suggest that phosphate uptake in this parasite is influenced by proton-dependent transport systems and may rely on coordinated interactions between distinct membrane transporters [27].

In trypanosomatids, phosphate uptake has also been shown to vary according to the parasite life cycle stage, closely correlating with proliferative capacity. In *Trypanosoma cruzi*, both H^+^-dependent and Na^+^-dependent phosphate transport systems were identified, mediated by transporters homologous to the yeast Pho84 and Pho89, respectively. Functional analyses revealed that epimastigote forms, which actively proliferate in the insect vector, display markedly higher Pi uptake rates compared to metacyclic trypomastigotes, the non-proliferative and infective stage [28]. Similarly, in *Leishmania amazonensis*, a high-affinity H^+^-dependent phosphate transporter encoded by the LamPho84 gene was characterized and shown to be differentially expressed throughout parasite differentiation. Promastigote forms exhibited significantly higher Pi transport activity and LamPho84 transcript levels than amastigote and metacyclic forms [29]. Together, these findings indicate that phosphate acquisition is preferentially enhanced in proliferative stages of trypanosomatid parasites, supporting increased biosynthetic and energetic demands, while infective forms display reduced phosphate uptake consistent with a metabolically quiescent state.

Evidence for stage-specific regulation of phosphate transport has also been reported in the free-living amoeba *Acanthamoeba castellanii*, further supporting a close association between Pi uptake and parasite developmental programs. In this organism, comparative analyses between trophozoites and cysts revealed a markedly higher phosphate uptake capacity in cyst forms, despite their classical description as metabolically quiescent stages. Molecular analyses identified multiple phosphate-transporter-related genes (AcPHS family) whose expression is differentially regulated across life stages, with specific isoforms preferentially expressed in cysts. Functional assays further demonstrated that cyst-associated phosphate transport systems display distinct kinetic and pH-dependent properties compared with those operating in trophozoites, consistent with the expression of different transporter repertoires. Importantly, enhanced Pi uptake in cysts was linked to anaerobic ATP synthesis pathways, indicating that phosphate acquisition remains metabolically relevant during encystment and contributes to cyst viability. Together, these findings challenge the traditional view of cysts as metabolically inactive forms and highlight phosphate transport as a critical component of stage-specific metabolic adaptation in protozoa [30].

Collectively, available evidence indicates that phosphate transport systems in protozoan parasites combine conserved mechanistic features with species-specific adaptations. While Na^+^-dependent (PiT-type) and H^+^-coupled (PHS-type) transporters are broadly distributed across taxa, their relative contribution appears to reflect the metabolic organization and environmental context of each organism. For instance, parasites with predominantly glycolytic metabolism, such as *Giardia* and *Tritrichomonas*, exhibit a tight functional coupling between phosphate uptake and ATP production, whereas in trypanosomatids, phosphate transport is more closely associated with developmental transitions and proliferative capacity. These differences suggest that phosphate acquisition strategies are not only conserved but also dynamically tuned to the physiological demands and ecological niches of each parasite.

Within this framework, the variability in phosphate acquisition strategies can also be understood in the context of host–parasite interactions. During infection, protozoan parasites rely on host-derived inorganic phosphate (Pi) while competing with host cells for this essential resource, often under conditions in which Pi availability is limited or unevenly distributed. Such environmental constraints likely contribute to the diversification of transport systems and the emergence of complementary acquisition mechanisms, including the ability to modulate transporter activity and to exploit alternative extracellular phosphate sources. In this sense, phosphate uptake is not only shaped by intrinsic metabolic demands but also by the selective pressures imposed by the host environment.

Despite species-specific differences in phosphate transport systems, most protozoan parasites analyzed to date share a common functional pattern: phosphate availability in the growth environment modulates phosphate uptake capacity. Although this regulation has not been experimentally evaluated in all species in which phosphate transport has been described [19,29,30], increased transporter activity under low-Pi conditions emerges as a recurrent observation across multiple protozoa, while changes in the expression of Pi transport genes appear to be species-dependent [20,21,22,23,24,25,26,27,28]. In *T. foetus*, evidence for phosphate-dependent regulation derives primarily from functional assays, in which phosphate limitation impacts ATP production and parasite growth, highlighting the requirement for sustained phosphate uptake to maintain cellular metabolism [22]. In *T. gondii*, parasites respond to phosphate deprivation by redistributing the TgPiT transporter from intracellular compartments to the plasma membrane, without significant alterations in gene expression, indicating a post-transcriptional mechanism of regulation [20].

## 4. Extracellular Phosphate-Releasing Activities: Enzymatic Strategies for Phosphate Scavenging

**Figure 2:**
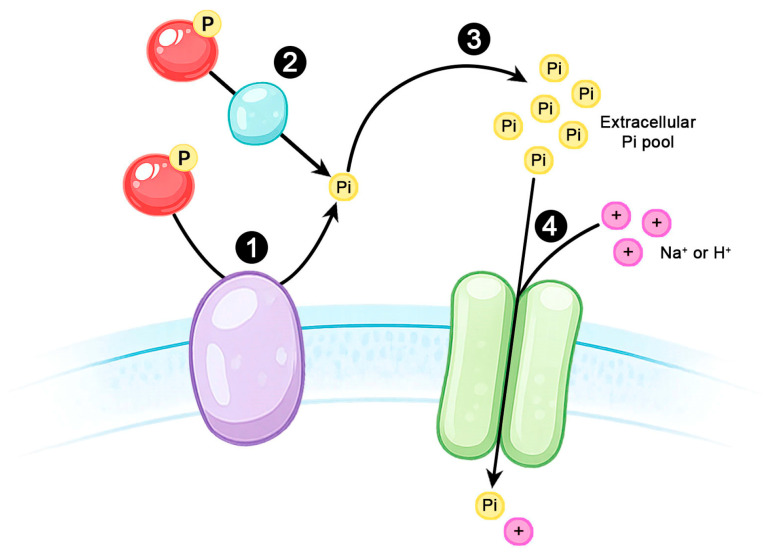
Conceptual model of phosphate acquisition under low-phosphate conditions in protozoan parasites. Red spheres labeled “P” represent generic extracellular phosphorylated substrates, including metabolites or proteins. Under low-Pi conditions, protozoan parasites exhibit adaptive responses characterized by an increased reliance on extracellular phosphate-scavenging activities. These include surface-associated ectophosphatases (1) and secreted phosphatases (2), which hydrolyze phosphorylated substrates and release inorganic phosphate (Pi) into the extracellular milieu (3). The liberated Pi is subsequently internalized via Na^+^- or H^+^-coupled phosphate transport systems (4). Under phosphate limitation, these transport systems display increased uptake capacity, resulting from enhanced transporter activity and, in some species, upregulated expression of Pi transporters. This schematic represents a conceptual framework integrating evidence from diverse protozoan lineages and does not imply the existence of a conserved molecular signaling pathway or direct regulatory mechanisms.

Phosphate-responsive phosphatase activities have been described in a wide range of microorganisms, including prokaryotes, parasitic protozoa, and fungi, indicating that regulated extracellular phosphate hydrolysis represents a conserved strategy for nutrient acquisition. By converting extracellular substrates into free inorganic phosphate, these enzymes expand the pool of available Pi and functionally couple phosphate scavenging to membrane transport systems. These strategies include the action of surface-associated and secreted phosphatases, as well as extracellular nucleotide-hydrolyzing enzymes, which collectively contribute to the hydrolysis of phosphorylated compounds in the surrounding milieu [31]. This functional integration between extracellular hydrolysis and phosphate uptake is schematically summarized in Figure 2.

Phosphate-dependent regulation of ectophosphatase activity has been experimentally demonstrated in several protozoan parasites [32,33,34]. Findings from studies in *Leishmania* and other medically significant protozoa support a critical role for these enzymes in parasite survival and infectivity [35,36,37]. In *T. rangeli,* ectophosphatase activity is enhanced under phosphate-depleted growth conditions, consistent with an adaptive response to phosphate limitation [38]. Similarly, in *T. foetus*, functional assays indicate increased reliance on ectophosphatase-mediated phosphate release under low-phosphate conditions, linking extracellular phosphate scavenging to intracellular ATP production and parasite growth [22].

Beyond ectophosphatases, additional enzymatic activities involved in extracellular phosphate release have also been shown to be modulated by phosphate availability in the growth environment. In *L. infantum*, parasites express a surface-associated 3′-nucleotidase whose activity is regulated by extracellular phosphate levels; this enzyme hydrolyzes 3′-AMP, generating adenosine and inorganic phosphate [39]. Similarly, in *T. rangeli*, an ecto-pyrophosphatase has been described in addition to classical ectophosphatase activity, catalyzing the hydrolysis of pyrophosphate into two molecules of Pi [40]. Notably, both activities are responsive to phosphate availability, as parasites grown under phosphate-limiting conditions exhibit higher enzymatic activity compared to those maintained in phosphate-replete media [39,40].

## 5. Functional Integration of Ectophosphatases and Phosphate Transport in Protozoan Parasites: Conceptual Models and Knowledge Gaps

Accumulating evidence indicates that protozoan parasites respond to phosphate limitation through coordinated adaptive mechanisms that include the modulation of both extracellular phosphate-scavenging activities and membrane phosphate transport systems. Rather than acting as independent processes, ectophosphatases, nucleotidases, and Pi transporters function as interconnected components of a broader phosphate acquisition strategy that supports parasite survival, growth, and differentiation in nutrient-restricted environments.

Across phylogenetically diverse protozoa, phosphate availability emerges not only as a metabolic constraint but also as a regulatory cue that shapes transporter activity, enzyme function, and, in some cases, stage-specific expression patterns. Despite marked differences in transporter families, ionic coupling mechanisms, and life cycle complexity, these organisms display a convergent functional logic in which extracellular phosphate hydrolysis and transmembrane uptake are dynamically adjusted to metabolic demand. This functional integration is particularly evident in parasites inhabiting fluctuating or phosphate-poor niches, where sustained phosphate acquisition is essential to maintain intracellular homeostasis, ATP production, and biosynthetic capacity.

An overview of protozoan species in which phosphate transporters and extracellular phosphate-scavenging enzymes have been described, together with the available evidence for phosphate-dependent regulation, is provided in Table 1. Importantly, although phosphate-responsive modulation of these processes has been demonstrated in multiple organisms, the molecular mechanisms underlying phosphate sensing and signal transduction in protozoan parasites remain largely unresolved. In contrast to the well-characterized PHO pathway in yeast and phosphate starvation responses in plants, protozoan-specific regulatory networks appear to be more diverse and context-dependent, highlighting substantial gaps in our understanding of how extracellular phosphate availability is perceived and translated into coordinated cellular responses.

## 6. Perspective/Future Outlook

Future studies integrating molecular genetics, biochemical approaches, and systems-level analyses will be essential to elucidate the signaling pathways that coordinate ectophosphatase activity, phosphate transport, and intracellular phosphate utilization in protozoan parasites. A deeper understanding of these regulatory mechanisms will not only advance fundamental knowledge of parasite metabolism and adaptation but may also uncover novel vulnerabilities that can be explored for therapeutic intervention.

## Figures and Tables

**Table 1:** Protozoan parasites exhibiting phosphate-dependent modulation of phosphate acquisition mechanisms.

Protozoan Parasites	Phosphate Transport Systems	Phosphate-Dependent Modulation
*Trypanosoma rangeli*	Na^+^-dependent and H^+^-dependent Pi transport systems	Transport activity increased under low Pi (without changes in *TrPHO89* gene expression) [23]; ectophosphatase [38] and ectopyrophosphatase [40] activities enhanced under low Pi
*Trypanosoma cruzi*	Na^+^-dependent and H^+^-dependent Pi transport systems	Transport activity increased under low Pi (without changes in *TcPHO89* and *TcPHO84* gene expression) [28]
*Trypanosoma brucei*	H^+^-dependent Pi transport system	Transport activity increased under low Pi (gene expression was not evaluated) [27]
*Leishmania infantum*	H^+^-dependent Pi transport system	Transport activity and *LiPHO84* gene expression increased under low Pi [24] Phosphate-dependent modulation of Pi-releasing surface 3′-nucleotidase activity [39]
*Leishmania donovani*	Na^+^-dependent and H^+^-dependent Pi transport systems	*LdPHO89* and *LdPHO84* gene expression increased under Pi deprivation [25]
*Toxoplasma gondii*	Na^+^-dependent Pi transport system	TgPiT is relocated to the plasma membrane under low Pi, without changes in gene expression [20].
*Giardia duodenalis*	H^+^-dependent Pi transport system	Transport activity and *GdPHO84* gene expression increased under low Pi [21]
*Phytomonas serpens*	Na^+^-dependent and H^+^-dependent Pi transport systems	Transport activity increased under low Pi for both systems; *PsPHO89* expression upregulated, *PsPHO84* expression unchanged [26]
*Tritrichomonas foetus*	Pi transport functionally coupled to ectophosphatase activity	Pi availability modulates ATP levels and parasite proliferation via ectophosphatase-mediated Pi acquisition (gene expression not evaluated); modulation occurs at the metabolic level [22]

This table summarizes protozoan species in which extracellular phosphate availability modulates inorganic phosphate (Pi) acqusition through regulation of membrane transport systems and/or extracellular phosphate-scavenging enzymes, such as ectophosphatases or nucleotidases. Evidence includes changes in transport activity, enzyme activity, gene expression, or functional outcomes under phosphate-limiting conditions.

## Data Availability

No new data were created or analyzed in this study. Data sharing is not applicable to this article.

## Abbreviations

The following abbreviations are used in this manuscript:

Pi	Inorganic Phosphate
PHO pathway	Phosphate signal-transduction pathway
